# Perceptual learning is robust to manipulations of valence and arousal in childhood and adulthood

**DOI:** 10.1371/journal.pone.0266258

**Published:** 2022-04-19

**Authors:** Aaron Cochrane, Ashley L. Ruba, Alyssa Lovely, Finola E. Kane-Grade, Abigail Duerst, Seth D. Pollak

**Affiliations:** 1 Faculty of Psychology and Educational Sciences, University of Geneva, Geneva, Geneva, Switzerland; 2 Department of Psychology, University of Wisconsin–Madison, Madison, Wisconsin, United States of America; 3 Institute of Child Development, University of Minnesota, Minneapolis, Minnesota, United States of America; 4 Homer Stryker M.D. School of Medicine, Western Michigan University, Kalamazoo, Michigan, United States of America; Yamaguchi University: Yamaguchi Daigaku, JAPAN

## Abstract

Despite clear links between affective processes in many areas of cognition and perception, the influence of affective valence and arousal on low-level perceptual learning have remained largely unexplored. Such influences could have the potential to disrupt or enhance learning that would have long-term consequences for young learners. The current study manipulated 8- to 11-year-old children’s and young adults’ mood using video clips (to induce a positive mood) or a psychosocial stressor (to induce a negative mood). Each participant then completed one session of a low-level visual learning task (visual texture paradigm). Using novel computational methods, we did not observe evidence for the modulation of visual perceptual learning by manipulations of emotional arousal or valence in either children or adults. The majority of results supported a model of perceptual learning that is overwhelmingly constrained to the task itself and independent from external factors such as variations in learners’ affect.

## 1. Introduction

Perceptual learning involves improvements in low-level sensory representations as well as the attentional and decision processes that convert such representations into actions [1–5]. These processes contribute to a range of complex behaviors that are important for typical development, such as face processing [6], reading [7], occupational expertise [8, 9], and higher cognition [5, 10]. Changes associated with learning are often the result of extensive experience [11–14] but may also occur quite quickly [2, 15] However, little is understood about how internal states (e.g., emotional valence or arousal) influence these learning processes, despite the established neural links between certain affective and learning processes (e.g., links between reward and learning networks, [16]; common stress neuromodulators of hippocampal and amygdalar function [17, 18]). Here, we combine perspectives from perceptual, affective, and developmental sciences to assess whether components of learning are influenced by manipulations of either valence (i.e., positive vs. negative mood manipulation) or arousal (manipulated vs. non-manipulated mood).

While perceptual learning has typically been considered independently of possible affective influences, and therefore tacitly assumed to be independent of mood, theories of perceptual learning do emphasize the possible role of affective factors (e.g., increased arousal in response to reward or brain stimulation [19]). Outside of learning studies positive and negative moods have been associated with distinct patterns of perception. Specifically, positive mood has been associated with broadening of the perceptive field and increased perception of global features rather than local features [20]. This may be understood as a “broadening and building” perceptual state, which is related to more creative, flexible cognition as well as a tendency towards holistic [21]. Such states may be related to superior learning (e.g., of language, [22]). Negative mood, on the other hand, may be associated with increased selective attention and narrowing of the perceptual field, which is associated with more goal directed, focused patterns of cognition (possibly impairing learning in the absence of well-established perceptual templates [23]).

While positive and negative valences would be expected to have opposite effects when considered on a single dimension, each manipulated valence would be expected to have the same effect when considered on the dimension of arousal (i.e., elevated arousal relative to baseline; see the “bipolar model of affect” [24]). Affective arousal may alter neuromodulator balances of noradrenaline and dopamine, thereby enhancing the perceptual encoding of events [25]. In their extremes, over-arousal and under-arousal are associated with poorer performance, leading to a curvilinear relationship between arousal and performance [26] (see [27] for discussion in the context of the misunderstood "Yerkes-Dodson Law").

Separating valence and arousal dimensions allows for the dissociation of these dimensions. For example, in studies assessing visual attention along both valence and arousal dimensions, increased negative-valence arousal was facilitatory while increased positive-valence arousal was detrimental [28]. Previous perceptual learning experiments with mood manipulations have been more limited, finding that interspersed negative-valence images facilitated learning in easy conditions, but not in hard conditions [29]. However, effects of mood on perceptual learning have not been probed along both arousal and valence dimensions in fully-crossed experiments, and development-related differences in such effects have been disregarded, despite clear lifelong benefits of successful perceptual learning (e.g., in reading [7]).

Prior investigations of affective influences on perceptual learning (or theoretical framework of [19, 29]) have also been limited by a reliance on aggregating perceptual performance at the timescale of blocks of trials. Learning as a continuous function of experience more accurately captures the underlying mechanism of change, as indicated by theories of learning [30–32] as well as by empirical studies [33, 34]. Thus, we were motivated to characterize the continuous changes in performance associated with learning in our perceptual learning task. In this task (i.e., the visual texture paradigm of Ahissar & Hochstein [3]), participants show relatively rapid decreases in the time necessary to detect an oddball stimulus amidst identical distractors. We implemented nonlinear generalized mixed-effects Bayesian models of performance to allow the simultaneous estimation of group- and participant-level perceptual thresholds. Contrasting these models provided the additional ability to estimate Bayes Factors for model selection.

By decomposing learning into components of continuous change, the current study not only tested overall effects of the manipulated affective dimensions (i.e., arousal or valence), but also the specific effects on starting performance (immediate effects of the manipulation prior to learning), rate of change (affective moderation of learning speed), and asymptotic performance (affective moderation of limits on performance after learning). We manipulated 8- to 11-year-old children’s and young adults’ mood using video clips (to induce a positive mood) or a psychosocial stressor (to induce a negative mood). Each participant next completed one session of a low-level visual learning task (visual texture paradigm). We then used novel computational methods to test the influences of valence manipulations or arousal manipulations on visual task performance. We did not find evidence for effects of mood manipulations on any component of the learning trajectories, and Bayesian model comparisons indicated uniform evidence against differences between mood conditions in adults.

## 2. Materials and methods

### 2.1. Participants

The study was conducted following American Psychological Association (APA) ethical standards and with approval of the institutional review board at the University of Wisconsin–Madison. A power analysis indicated that a sample size of 28 participants per age/condition would be sufficient to detect reliable differences in a between-subjects design, assuming a medium effect size (*f* = .25, α = .05, power = .80). This was preselected as the target sample size for the study (given a lack of clear method for conducting a power analysis for our primary modeling approach).

Recruitment occurred in a medium-sized Midwestern city in the United States. Adults were recruited through an undergraduate psychology course subject pool, and children were recruited from the community. The adult sample included 86 students (53 females, *M*_*age*_ = 19.62 years, *SD*_*age*_ = 1.31 years, range = 18.10–25.31 years). Adults identified as White (74%, *n* = 64), Asian (15%, *n* = 17), multi-racial (2%, *n* = 2), Black (2%, *n* = 2), and Native American (1%, *n* = 1). Seven adults (8%) identified as Hispanic or Latino. The child sample included 86 8- to 11-year-old children (34 females, *M*_*age*_ = 9.14 years, *SD*_*age*_ = .79 years, range = 7.97–11.00 years). Children were identified as White (79%, *n* = 68), multi-racial (9%, *n* = 8), Asian (5%, *n* = 4), or Black (7%, *n* = 6). Nine children (10%) were identified as Hispanic or Latino. For their participation, adults were given course credit, and children received a small prize. Nine additional participants were tested but excluded for failure to finish the experiment (*n* = 7 children) or computer error (*n* = 2, one adult, one child).

### 2.2. Design

Adults and children were randomly assigned to one of three conditions: positive, negative, or control. All participants (except children in the control condition, see below) completed a fifteen minute “mood-induction” activity prior to the first block of the learning task. Between the first and second blocks of the learning task, child participants completed a three minute “mood-booster” activity. We added a mood-booster for the child participants as pilot testing indicated that (a) children were relatively slow to complete the perceptual learning task, and thus, (b) effects of the mood-induction seemed to degrade over the first block of the task.

#### 2.2.1. Adult participants

For the *positive mood-induction*, adults watched a series of short videos (approximately two to four minutes in length), selected from a researcher-provided list. At the end of fifteen minutes, adults selected which video they “liked the best,” and the experimenter praised the choice (e.g., “That’s a great video. I really like that one too.”). For the *stress mood-induction*, adults completed the Trier Social Stress Test (TSST) [35]. This is a common, well-validated method of inducing negative stress (see S1 File for full description). For the *control (neutral) mood-induction*, participants completed a fifteen-minute computer game. In the game, a cluster of red dots appeared in one location on the screen. Participants clicked on the screen to indicate where they believed the cluster would move. The dot clusters moved in semi-random patterns, and participants were given written feedback about how correct their guess was (e.g., “you guessed X% of the cluster’s movement”).

#### 2.2.2. Child participants

For the *positive-mood induction*, children watched a series of short videos (approximately two to four minutes in length), selected from a researcher-provided list (including the same videos provided to adult participants). At the end of fifteen minutes, children selected which video they “liked the best,” and the experimenter praised the choice (e.g., “That’s a great video. I really like that one too.”). For the *positive mood-booster*, children selected one additional video to watch from the provided list. For the *stress-mood induction*, children completed a video-based version of the Trier Social Stress Test for Children (TSST-C) [36, 37] (see S1 File for full description). Child participants in the *control condition* did not complete a mood-induction. Pilot testing revealed that children reported negative mood ratings (i.e., due to “boredom”) following any fifteen minute “neutral” activity (e.g., the same computer game used for adult controls, watching nature videos, etc.). For the *control mood-booster*, children took a three-minute walk or stretch break.

### 2.3. Measures

#### 2.3.1. Perceptual learning task

All participants completed a computer-based visual learning task (texture oddball detection [38]). In this task, participants viewed a 7 x 7 grid of oriented lines. In half of the trials, all lines had the same orientation. In the other half of the trials, one line was offset by 30°. Participants saw the grid at randomly distributed stimulus onset asynchronies (SOAs) of 15, 30, 60, 90, 120, 300 or 500ms, followed by a pattern masking of the grid. For each trial, participants used one of two marked keys to indicate whether the lines all had the same orientation or if the grid included an offset line. Participants completed two blocks of 210 trials.

Learning in a perceptual task on this timescale is more abbreviated than most perceptual learning studies (e.g., those that train participants over thousands of trials and multiple sessions, [3, 14, 39]). Indeed, we make no claims about the locus of change occurring in our learning task and timeframe, and our observed improvements in performance may be attributed to attentional allocation, noise suppression, texture perception, or a combination of such candidate loci of change. We instead use the label “perceptual learning” as an expedient name for our learning task that conforms both with the trial structure, task demands [38, 40] and the timescale [1, 2, 41, 42] of previous research that has used the label “perceptual learning.”

#### 2.3.2. Mood ratings

The Self-Assessment Manikin (SAM) [43] was used to assess participants’ mood at three time-points throughout the experiment. The SAM contains five pictorial depictions of mood, ranging from a smiling figure (i.e., very positive) to a frowning figure (i.e., very negative). Participants selected one figure in each category to indicate their current mood.

### 2.4. Procedure

After obtaining informed consent (adult participants) or informed parental consent and child assent (child participants), all participants completed the first SAM (Time 1 –start of study). Participants (except for the *child control condition*) completed mood-induction activities. After the mood-induction, participants completed the second SAM (Time 2 –after mood induction) and the first block of the perceptual leaning task. Children in the control condition proceeded directly from the first SAM to the first block of the perceptual learning task. For these children, their ratings for the second SAM are identical to their ratings on the first SAM. After the first block, all participants either took a three-minute break (*child control condition* and all adults) or completed a mood-booster activity: watching videos (*child positive condition*) or math problems (*child stress condition*). This was followed by the second block of the perceptual learning task and the final SAM (Time 3 –end of study).

### 2.5. Analytical approach

Continuous changes in performance were modelled using generalized mixed-effects models in which Weibull [Quick] psychometric function (PF [3, 44–46]) threshold was fit as a nonlinear exponentially-saturating function of trial number [34, 47–49]. All models were fit using the R package **brms** [50]. Model details and code are included in S1 File. In summary, these models characterized accuracy for each trial for each participant as a function of SOA, using an adapted cumulative Weibull function that interpolated between chance performance (50%) and perfect performance with a small lapse rate [38, 40]. This psychometric function was then estimated in a hierarchical Bayesian model framework in which the threshold (i.e., the SOA associated with 75% accuracy) changed as an exponential function of increasing task experience [40, 49] (see S1 File for plots of group-averaged PF over time). A lower threshold is indicative of superior performance, as it means that the task can be completed accurately with a smaller SOA. The other parameter in the Weibull PF (shape) was estimated as being constant over time for each participant. Components of change (starting threshold, time taken to change, and asymptotic threshold) as well as the PF shape were each simultaneously estimated in generalized multilevel models including main effects of condition (i.e., valence and arousal) and by-participant random effects. That is to say, each of these components of change could be independently influenced by mood manipulations, and could indicate different possible routes by which learning would be altered by valence or arousal (i.e., by initial differences, differences in rate of learning, or by alterations in the best performance reached after extensive learning [51]).

Two methods were used to interpret model results. Conventional parameter reliability was determined by assessing each parameter’s 95% CI in relation to zero. Model comparison and selection utilized Bayes Factors estimated using **brms**.

## 3. Results

### 3.1. Manipulation check

**Fig 1:**
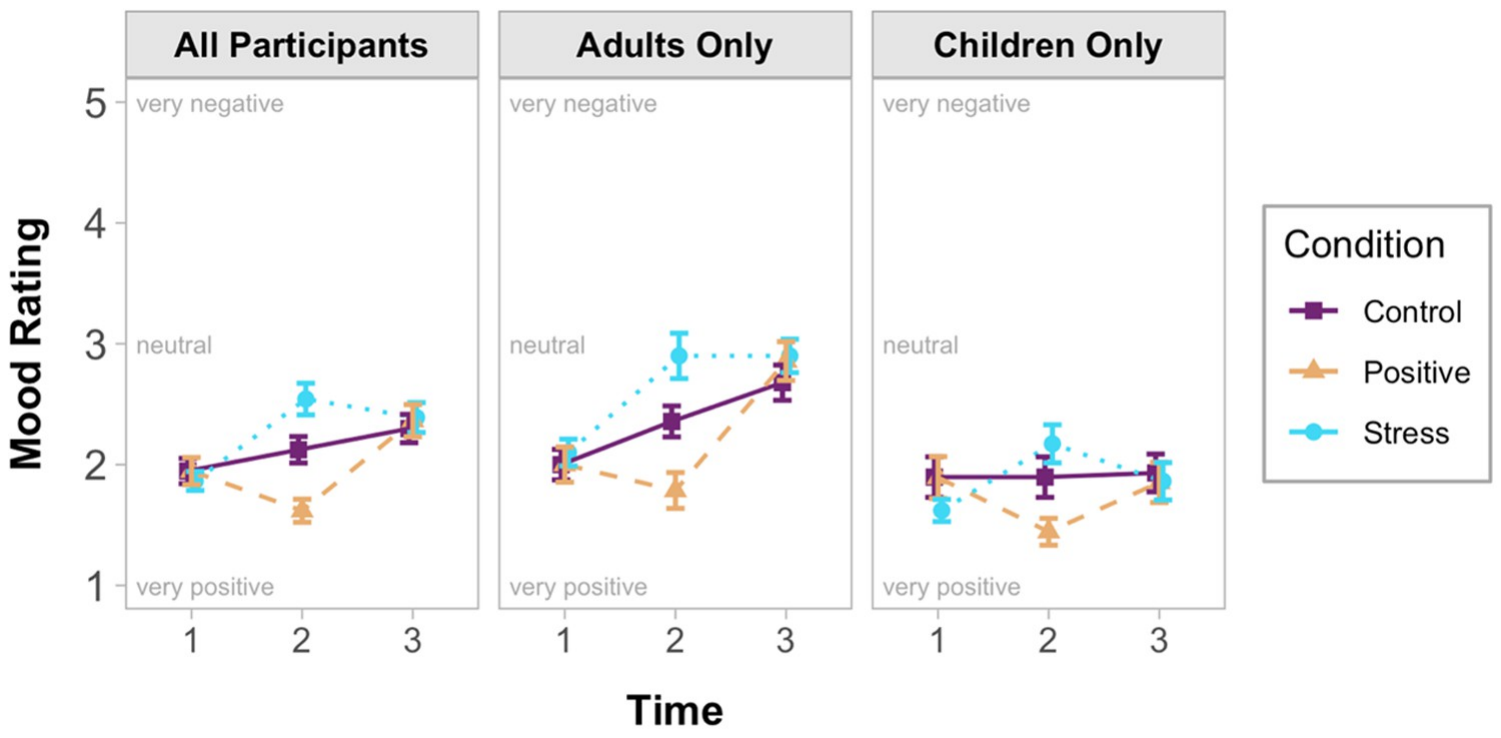
Participant self-reported mood ratings separated by condition, time, and participant age.

To check the effectiveness of the mood induction, participants’ SAM ratings were analyzed in a 3 (Time: T1 / T2 / T3) x 3 (Condition: positive / stress / control) x 2 (Age: child / adult) mixed-methods ANOVA. A Time x Condition interaction emerged, *F*(4, 303) = 13.75, *p* < .001, *η*_*p*_^*2*^ = .14 (Fig 1). Follow-up ANOVAs showed that mood ratings did not differ by Condition for T1 (start of study), *F*(2, 166) = .27, *p* > .25, *η*_*p*_^*2*^ < .01, or T3 (end of study), *F*(2, 165) = .13, *p* > .25, *η*_*p*_^*2*^ < .01. As hypothesized, there was a significant effect of Condition at T2 (after mood-induction), *F*(2, 165) = 17.93, *p* < .001, *η*_*p*_^*2*^ = .18. Mood ratings in the stress condition were significantly more negative than mood ratings in the control condition, *t*(114) = 2.45, *p* = .016, *d* = .47, CI_95%_[.08, .76], and the positive condition, *t*(112) = 5.64, *p* < .001, *d* = 1.06, CI_95%_[.60, 1.25]. Mood ratings were significantly more positive for the positive condition compared to the control condition, *t*(110) = 3.37, *p* < .001, *d* = .66, CI_95%_[.22, .79]. There were no significant Interactions with Age.

Additional comparisons were conducted to confirm that participants’ mood ratings significantly changed following each mood-induction. Compared to T1 (start of study), mood ratings at T2 (after mood-induction) were significantly more positive in the positive condition, *t*(54) = 3.66, *p* < .001, *d* = .49, CI_95%_[.16, .53], and significantly more negative in the stress condition, *t*(58) = 6.36, *p* < .001, *d* = .83, CI_95%_[.46, .89]. Together, these analyses confirm the success of the mood-induction activities.

### 3.2. Preliminary description of performance

Various participants, particularly children, had difficulty completing the task at above-chance performance levels. We identified participants to exclude by testing whether, over all trials, performance was significantly above 50% (i.e., one-tailed binomial *p* value < .05). We were left with a final sample of 78 adults and 68 children (see Table 1). The pattern of exclusions did not differ by experimental condition or by participants’ mood ratings at T1 or T2 (see S1 File for further details).

**Table 1:** Sample sizes, for each age group and condition, after excluding participants for chance performance on the learning task.

	Control	Positive	Stress
Adults	26	25	27
Children	21	22	25

**Fig 2:**
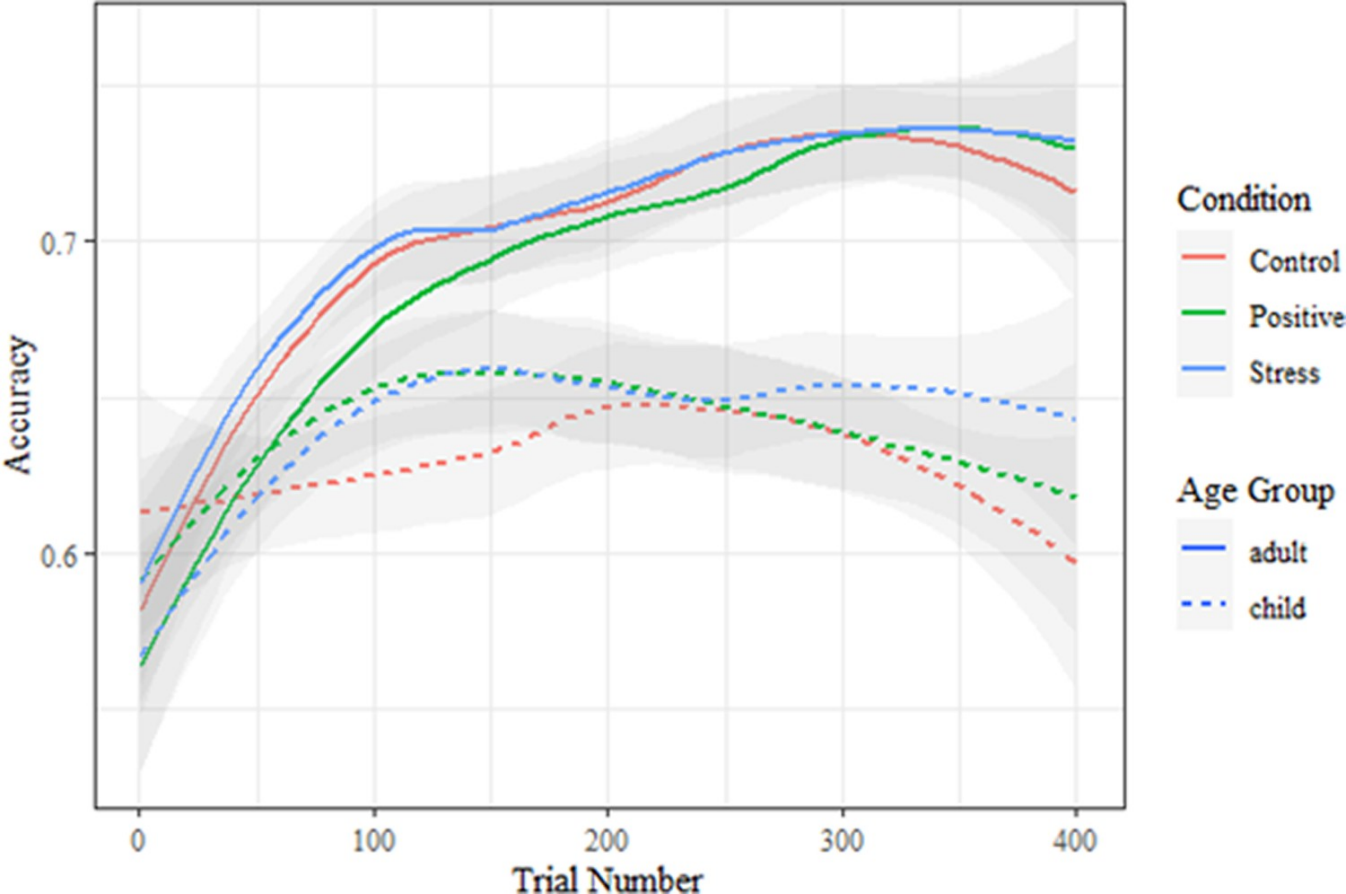
LOESS-smoothed accuracies for each experimental group over the course of the training.

We next examined the overall effects by visualizing the raw accuracies of participants in each condition (see Fig 2). Due to the apparent decrement of performance in later trials, and in the interest of maximizing our ability to detect differences due to the mood induction (which occurred before trial 1), we chose to apply learning models to only the first 200 trials (for models fit to all trials, with qualitatively the same results, see S1 File). Note that differences between conditions in Fig 2 should not be interpreted as indicative of true underlying group differences due to the model-free smoothing involved (i.e., the following model-based analyses of learning are less likely to be influenced by idiosyncrasies of certain participants or of the LOESS smoothing algorithm [52]).

### 3.3. Overall model

**Fig 3:**
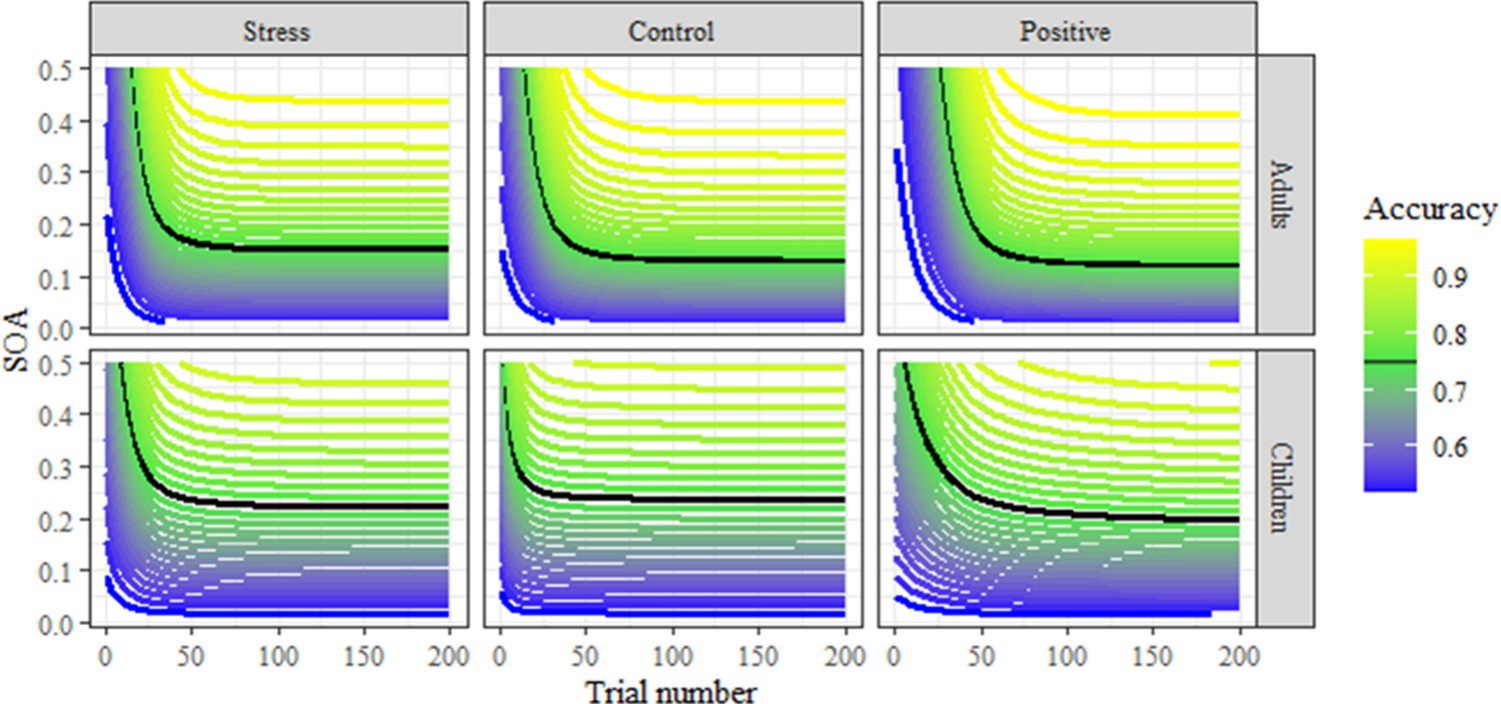
Group-level learning curves of the full model fitting monotonic effects of arousal as well as binary effects of age and valence. Contour lines represent levels of accuracy, with smaller SOA for a given accuracy indicating superior performance (i.e., contours lower on the Y axis indicate equal performance at shorter presentation times). The fit threshold value (75%) is indicated as a black contour, with a decrease over time in threshold evident in each group (i.e., learning).

We first report the results of a model fitting the first 200 trials of both age groups and all experimental conditions (see Fig 3 and Table 2). Arousal was coded as -.5 (Control) and +.5 (Stress and Positive), while valence was modeled as a monotonic (but not necessarily linear; see [53]) effect with the Control condition being placed between the Stress and Positive conditions. This model did not indicate any reliable main effects of arousal or valence, or any interactions with age. The one reliable effect evident from this model was lower asymptotic thresholds reached by adults. We also tested for interactions between emotion dimensions in an analogous model including three-way interactions between valence, arousal, and age groups. This more complex model also did not have any reliable main effects of or interactions involving arousal or valence, and we chose to report the more parsimonious model here.

**Table 2:** Coefficients and CI from the full mixed-effects model of exponential learning.

	Estimate	lower 95% CI	upper 95% CI	reliable
Asym: Intercept	-2.381	-2.685	-2.040	
Asym: age	-0.639	-1.082	-0.159	*
Asym: arousal	-0.108	-0.504	0.247	
Asym: age × arousal	0.232	-0.344	0.798	
Asym: valence	-0.132	-0.361	0.082	
Asym: age × valence	-0.054	-0.412	0.318	
Start: Intercept	0.584	-0.288	1.591	
Start: age	0.988	-0.242	2.225	
Start: arousal	0.553	-0.625	1.672	
Start: age × arousal	0.695	-0.630	2.060	
Start: valence	0.013	-0.720	0.862	
Start: age × valence	0.747	-0.313	1.851	
Rate: Intercept	2.156	0.968	3.459	
Rate: age	0.502	-1.889	2.614	
Rate: arousal	0.705	-1.020	2.406	
Rate: age × arousal	-1.319	-4.081	1.778	
Rate: valence	0.418	-0.500	1.236	
Rate: age × valence	-0.330	-1.787	1.101	
PF shape: Intercept	1.173	1.099	1.257	

Due to the lack of reliable effects in the full model of both age groups and affective dimensions, we concluded that there were no clear conditions differences in learning shared by both ages. There were likewise no clear differences between age groups in learning due to valence or arousal manipulations. We also tested the models’ fit magnitudes of learning (i.e., each participant’s difference between starting and ending accuracy, evaluated at the mean SOA), and no effects of arousal or valence were reliably different than zero in either children or adults.

For the purposes of understanding the two manipulation dimensions (and quantifying the evidence using Bayes Factors) in each age separately, we ran one nonlinear generalized mixed-effects model for each combination of age group and affective manipulation. Models were specified as described above (and see the S1 File), with several key changes. Age groups were fit separately to identify age-specific patterns in learning, thereby removing the need to estimate the effect of age on learning parameters. Arousal and valence models were likewise fit separately. When testing valence effects, only Stress and Positive conditions were included (i.e., unlike in the full model, valence was not modeled as a three-level monotonic effect). Each model therefore characterized improvements in performance as exponentially decreasing Weibull PF thresholds, with the parameters of the exponential change each being predicted by a two-group main effect of affective manipulation as well as by-participant random effects. In order to compute Bayes Factors we fit a corresponding set of four models without the affective manipulation main effects, and were thereby able to use bridge sampling model comparisons [54] to estimate the relative evidence for the effects of the affective dimensions. We report the base-3 log of the Bayes Factor due to its simple interpretation regarding hypothesis testing (i.e., using conventional thresholds, below -1 indicates at least moderate evidence for the simpler model, while above 1 indicates at least moderate evidence for the more complex model).

### 3.4. Manipulations of valence on perceptual learning

We first tested the extent to which perceptual learning in children may be influenced by emotional valence manipulations. No reliable effects of valence were found (see Table 3). Model comparison indicated strong support for the lack of a valence-dependent modulation of learning (log_3_BF = -3.33).

**Table 3:** Coefficient and CI from model testing valence-manipulation effects in children.

	Estimate	lower 95% CI	upper 95% CI
Asym: Intercept	-2.05	-2.33	-1.74
Asym: valence	0.10	-0.38	0.59
Start: Intercept	-0.19	-1.07	0.98
Start: valence	0.16	-1.11	1.51
Rate: Intercept	3.16	1.54	4.57
Rate: valence	-0.03	-2.25	2.15
PF shape: Intercept	1.20	1.09	1.33

We next tested whether learning in adults was influenced by valence manipulations. No reliable effects of valence were found (see Table 4). Model comparison indicated strong support for the lack of a valence-dependent effects on learning (log_3_BF = -2.92).

**Table 4:** Coefficient and CI from model testing valence-manipulation effects in adults.

	Estimate	lower 95% CI	upper 95% CI
Asym: Intercept	-2.51	-2.91	-2.06
Asym: valence	0.06	-0.49	0.59
Start: Intercept	1.52	0.54	2.62
Start: valence	0.45	-0.86	1.77
Rate: Intercept	2.66	1.95	3.36
Rate: valence	0.68	-0.56	1.90
PF shape: Intercept	1.08	0.96	1.24

### 3.5. Manipulations of arousal on perceptual learning

Next, we assessed the degree to which children’s perceptual learning was modulated by emotional arousal manipulations. No reliable effects of arousal were found (see Table 5). Model comparison indicated equivocal support for the lack of an arousal-dependent variations in learning (log_3_BF = -0.45). This small absolute Bayes Factor indicates similar evidence for the model including arousal effects and the model without arousal effects.

**Table 5:** Coefficient and CI from model testing arousal-manipulation effects in children.

	Estimate	lower 95% CI	upper 95% CI
Asym: Intercept	-2.04	-2.25	-1.83
Asym: arousal	-0.16	-0.54	0.20
Start: Intercept	-0.31	-1.14	0.79
Start: arousal	0.06	-1.24	1.29
Rate: Intercept	2.52	1.13	3.91
Rate: arousal	1.71	-0.37	3.60
PF shape: Intercept	1.29	1.18	1.40

Last we tested whether perceptual improvements in adults were influenced by arousal manipulations. No reliable effects of arousal were found (see Table 6). Model comparison indicated moderate support for the lack of an arousal-dependent modulation of learning (log_3_BF = -1.46).

**Table 6:** Coefficient and CI from model testing arousal-manipulation effects in adults.

	Estimate	lower 95% CI	upper 95% CI
Asym: Intercept	-2.77	-3.10	-2.40
Asym: arousal	0.00	-0.47	0.49
Start: Intercept	1.37	0.46	2.39
Start: arousal	0.85	-0.42	2.12
Rate: Intercept	2.66	1.88	3.46
Rate: arousal	0.01	-1.30	1.36
PF shape: Intercept	1.09	0.99	1.21

## 4. Discussion

For both children and adults, we did not observe clear evidence for the modulation of visual perceptual learning by manipulations of emotional arousal or valence. We estimated by-trial changes in performance using nested Bayesian hierarchical nonlinear models of change in psychometric thresholds. The resulting Bayes Factors indicated a uniform evidence for a lack of affect-related influences on perceptual learning in adults. In contrast, when testing the results of the mood manipulation on children, strong evidence was found for a lack of valence-related effects, while the effects of arousal on learning were equivocal.

One direct implication of these results is that, given the evidence against influences of arousal or valence on adults’ performance, the *status quo* assumptions of much of cognitive and perceptual psychology appears vindicated. That is, in our adult sample, the lack of mood-based effects appears to justify the paradigmatic disregard for affective measures when studying visual perception and attention. Especially in the field of visual perceptual learning, where prior research at this intersection has been nearly nonexistent, our result provides initial evidence to confirm the tacit assumption of the field. Our results only directly inform learning in a low-level visual task on a fairly short timescale, which may differ from learning on a longer timescale (e.g., as in most perceptual learning experiments) or with more complex stimuli. The field of perceptual learning itself quite heterogeneous and studies with a variety of training protocols and extended timescales would provide even stronger inferences regarding the robustness of learning to variations in valence or arousal. Yet to the extent that common processes are involved, it seems that our lack of mood manipulations’ effects remains congruent with the dominant assumptions of related fields of research.

Perhaps more importantly, the robustness of learning we observed carries encouraging implications for learners across the lifespan. Given the many day-to-day activities that are supported by learning to tune perceptual discriminations and decisions, negative effects on learning of negative mood could hypothetically cause a detrimental developmental cascade. To the contrary, our results indicate that children’s and adults’ learning are each resilient to external manipulations of mood. Our conclusions in this regard are very preliminary and the generalization of our results is limited (see below), yet we believe that these results still provide grounds for optimism.

These implications come with two important caveats. First, the evidence for null effects was not uniformly present in children’s data. While there was compelling evidence against valence effects on learning-task performance, the evidence regarding arousal effects was equivocal. Thus, strong inferences are not justified. Instead, the present study indicates that additional investigation is necessary to clarify the potential influences of arousal on children’s visual performance and learning. Further, the lack of conclusive evidence in children indicates that other populations that diverge from the high-functioning adult populations, such as those with various psychopathologies, may likewise have susceptibilities to arousal that diverge from the relative resilience of high-performing adults’ performance. Special populations would benefit from targeted studies of the affective influences on perceptual and attentional learning.

Second, we recognize that our inferences regarding mood effects were limited to only the single manipulation we implemented. It is certainly possible that alternative manipulations could lead to clear changes in components of visual performance and learning, and additional experimentation would be necessary to explore this possibility. In fact, several alternative explanations present themselves. The first concerns the efficacy of our mood manipulations. If the mood change induced by our manipulations was insufficient, then no effect on perceptual learning would be possible. This seems unlikely given that both mood manipulations had the predicted effects on self-reported moods immediately after the manipulation. Further, for our stress induction, the TSST is a common and well-validated method, and should produce a stronger negative affect than previously-implemented methods (e.g., interleaved emotional images [29]). Another possibility is that the effects of the mood manipulations rapidly decayed after the perceptual learning task started. This could explain differences between our results and interleaved-image studies, as well as providing one interpretation of the differences between children and adults (i.e., because children had a “booster” intervention). In the current study, we were unable to systematically test the rate of decay in the effects of the mood manipulation. Nonetheless, we believe that only additional studies, using a variety of mood manipulations and learning tasks, would be able to provide decisive evidence regarding the robustness of visual learning to variations in affect.

Our results support a model of learning in low-level visual processing that is overwhelmingly constrained to the task itself and independent from external factors such as variations in affect. These patterns held, for the most part, across development. While such conclusions support the robustness of previous models of perceptual learning, the equivocal results in children’s learning and the possibility of rapidly-decaying emotional manipulations should motivate further research at this intersection of development, perception, and learning.

## Supporting information

S1 File Supporting information includes additional descriptions of the behavioral methods, model specifications, patterns of participant exclusions, and alternative model fits that used data from all experimental trials. (DOCX)
