# Supplementary material for: Perceptual learning is robust to manipulations of valence and arousal in childhood and adulthood
Source: PLoS One. 2022 Apr 19;17(4):e0266258. doi: 10.1371/journal.pone.0266258 (PMC9017894; doi:10.1371/journal.pone.0266258)
Supplement: S1 File — (DOCX) [file pone.0266258.s001.docx]

**Supporting Information**

Perceptual Learning is Robust to Manipulations of Valence and Arousal in Childhood and Adulthood

## S1. Description of Stress Induction

### **S1.1. Adults – Trier Social Stress Test (TSST)**

Participants were told that they would be giving a five-minute speech in which they would describe why they are the best candidate for their ideal job. Participants were given ten minutes to prepare their speech in an isolated laboratory room. After the preparation phase, participants were led to a large conference room where three research assistants posed as “trained specialists in verbal and nonverbal communication.” The participant was instructed to stand in the center of the room, facing the “judges” and a camera. Participants were told that they were being recorded and that a voice frequency analysis of nonverbal behavior would be performed. Participants had five minutes to give their speech and were told that they should speak for the entire five-minute period. If the participant stopped talking for more than 20-s, they were prompted to continue speaking (i.e., “You still have time remaining.”). Following the speech task, participants completed a five minute math task in which they sequentially subtracted the number 13 from 1,023 out loud. If the participant made a mistake during this task, they were asked to start over from 1,023 (i.e., “That is incorrect, start over from 1,023”).

### **S1.2. Children – Trier Social Stress Test for Children (TSST-C)**

Children were told that they would be giving a five-minute speech in which they would finish a story based on a prompt. After the experimenter read the prompt, children were given three minutes to prepare their speech in an isolated laboratory room. After the preparation phase, the experimenter returned with a laptop and introduced participants to their peer judges via computer in a video format (Smith et al., 2020). Through scripted and timed interactions, participants watched the experimenter interact with each judge, leading participants to believe the judges were “live.” In fact, the child judges were actors who had been previously filmed within our laboratory. After the staged introductions, participants were told that the experimenter was going to transfer the judges’ video connection to a main computer so that the judges could watch the participant’s speech. Participants saw videos of the four judges who maintained neutral facial expressions with no visual or verbal feedback throughout the speech. Children had five minutes to give their speech and were told that they should speak for the entire five-minute period. If the participant stopped talking for more than 20-s, the experimenter gave prompts to continue speaking (e.g., “Expand the story. Be creative”). Following the speech task, children performed a five minute math task (i.e., subtracting the number 7 from 758). For the stress mood-booster, children completed three minutes of the same math task with the videos of the child judges brought back up on the screen.

## S2. Model Details and Code

Model formula:

Time-evolving psychometric function:

$\theta= .5+ .495\left( 1-{{2.0204}^{-\frac{SOA}{{asymptote+(start-asymptote)}^{\frac{1-trialNumber}{rate}}}}}^{PFshape} \right)$

Where *start*, *rate,* & *asymptote* are parameters defining the change in threshold (“location,” on the SOA scale) as a function of trial number, and each of them is estimated on a binary-log scale [link function] due to their natural bounds on positive reals. *PFshape* is the shape, or slope, of the Weibull psychometric function.

Parameter-level linear mixed-effects models:

$$start= X_{w_{s}}+Z_{u_{s}}$$

$$rate= X_{w_{r}}+Z_{u_{r}}$$

$$asymptote= X_{w_{a}}+Z_{u_{a}}$$

$$shape= Z_{u_{h}}$$

Where each $X_{w_{i}}$ is a fixed-effects structure including the main effects of zero-centered age, zero-centered arousal, monotonic effect of valence, and interactions between age and arousal as well as between arousal and valence. Each $Z_{u_{i}}$ is a random-effects structure including a participant-level intercept for each parameter. As described in the manuscript, reduced models were also run (e.g., removing the effects of age when fitting models to only children or only adults).

Model formula (**brms**):
>> acc ~ 0.5 + ((1 - 0.5) - 0.005) * (1 - 2.0204^(-(SOA/((2^(threshAsym) + (2^(threshStart) - 2^(threshAsym)) * 2^((1 - trialNum)/(2^(threshRate))))))^weibullshape))
>> threshAsym ~ ageC * arousal + ageC * mo(valence) + (1 || subID)
>> threshStart ~ ageC * arousal + ageC * mo(valence) + (1 || subID)
>> threshRate ~ ageC * arousal + ageC * mo(valence) + (1 || subID)
>> weibullshape ~ (1 || subID)

As is evident in the model formula, all parameters are estimated on a binary-log scale to allow unrestricted sampling while limiting thresholds to positive values. The Weibull psychometric function was parameterized to start at 50% (i.e., chance), saturate to 99.5% (i.e., perfect accuracy with a lapse rate of .005), and have a stationary shape parameter across all trials. The threshold estimated was at 75% correct, as defined by the logarithm base of the PF (2.0204). A Bernoulli response distribution (i.e., objective function) was utilized. Priors were default/minimally-informative. Models were each run for 3 chains of 25,000 iterations each, with a warm-up period of 12,500 iterations and a thinning interval of 5 iterations.

##

## S3. Participant Exclusion

Although more children were excluded than adults, there were no reliable patterns across ages in excluded participants (chi-square = 0.38, *p*_Monte-Carlo_ = 1). Likewise, there were no reliable patterns in condition groups of the retained participants (in a model with both ages chi-square = 0.12, *p*_Monte-Carlo_ = 0.976; adults-only chi-square = 0.00, *p*_Monte-Carlo_ = 1; children-only chi-square = 1.78, *p*_Monte-Carlo_ = 0.41). When including all participants and testing for condition differences in overall accuracy, there were no condition pairwise differences in either adults (all Tukey HSD *p* > .79) or in children (all Tukey HSD *p* > .12).

In tests whether mood ratings were different between participants who were to be excluded or not, we found no differences. In linear models predicting initial mood ratings using exclusion status, condition (with neutral as reference), and their interactions, all exclusion status main effect and interaction coefficient *p* values were above .25 (adults) and above .11 (children). In related models predicting post-manipulation “time 2” mood ratings using exclusion status, condition (with neutral as reference), and their interactions, while controlling for initial mood ratings, the main effect and interactions involving exclusion status all had *p* values above .17 (adults) and above .14 (children). We note that, even if there were to be statistically reliable results, each of these models is likely to be difficult to interpret due to very unequal sample sizes (over 4:1 ratio in all conditions) between retained and excluded participants.

The overall pattern of these results indicate that participant exclusions were likely to be independent of both experimental condition and mood ratings.

##

## S4. Analysis of All Trials

When examining the overall averages per condition (i.e., Fig.2), there appeared to be a drop-off in performance toward the end. Also, not all participants completed identical numbers of trials. For these reasons, in the main manuscript we reported models fit to only the first 200 trials (see Figure S1). Here we report results of models fit to all trials, but otherwise remaining identical to the models reported in the main manuscript. As in the main manuscript, arousal and valence manipulations did not reliably influence the trajectories of learning (see Tables S2-S4).


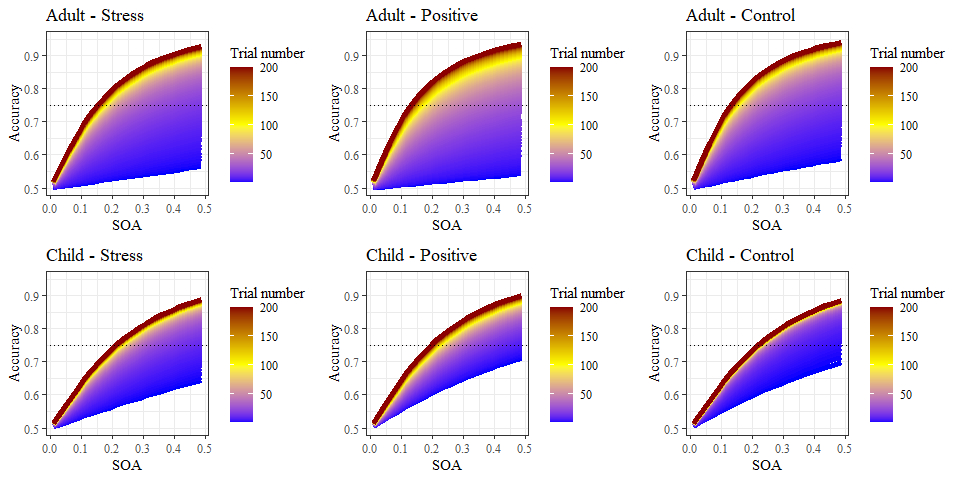


*Figure S1. Change in the Weibull psychometric functions as a continuous effect of time, divided by age group and affective manipulation.*

|  | **Estimate** | **lower 95% CI** | **upper 95% CI** |
| --- | --- | --- | --- |
| Asym_Intercept | -2.05 | -2.33 | -1.74 |
| Asym_valence | 0.10 | -0.38 | 0.59 |
| Start_Intercept | -0.19 | -1.07 | 0.98 |
| Start_valence | 0.16 | -1.11 | 1.51 |
| Rate_Intercept | 3.16 | 1.54 | 4.57 |
| Rate_valence | -0.03 | -2.25 | 2.15 |
| weibullshape_Intercept | 1.20 | 1.09 | 1.33 |

*Table S1. Coefficient and CI from model testing valence-manipulation effects in children, using all experimental trials.*

|  | **Estimate** | **lower 95% CI** | **upper 95% CI** |
| --- | --- | --- | --- |
| Asym_Intercept | -2.51 | -2.91 | -2.06 |
| Asym_valence | 0.06 | -0.49 | 0.59 |
| Start_Intercept | 1.52 | 0.54 | 2.62 |
| Start_valence | 0.45 | -0.86 | 1.77 |
| Rate_Intercept | 2.66 | 1.95 | 3.36 |
| Rate_valence | 0.68 | -0.56 | 1.90 |
| weibullshape_Intercept | 1.08 | 0.96 | 1.24 |

*Table S2. Coefficient and CI from model testing valence-manipulation effects in adults, using all experimental trials.*

|  | **Estimate** | **lower 95% CI** | **upper 95% CI** |
| --- | --- | --- | --- |
| Asym_Intercept | -2.04 | -2.25 | -1.83 |
| Asym_arousal | -0.16 | -0.54 | 0.20 |
| Start_Intercept | -0.31 | -1.14 | 0.79 |
| Start_arousal | 0.06 | -1.24 | 1.29 |
| Rate_Intercept | 2.52 | 1.13 | 3.91 |
| Rate_arousal | 1.71 | -0.37 | 3.60 |
| weibullshape_Intercept | 1.29 | 1.18 | 1.40 |

*Table S3. Coefficient and CI from model testing arousal-manipulation effects in children, using all experimental trials.*

|  | **Estimate** | **lower 95% CI** | **upper 95% CI** |
| --- | --- | --- | --- |
| Asym_Intercept | -2.77 | -3.10 | -2.40 |
| Asym_arousal | 0.00 | -0.47 | 0.49 |
| Start_Intercept | 1.37 | 0.46 | 2.39 |
| Start_arousal | 0.85 | -0.42 | 2.12 |
| Rate_Intercept | 2.66 | 1.88 | 3.46 |
| Rate_arousal | 0.01 | -1.30 | 1.36 |
| weibullshape_Intercept | 1.09 | 0.99 | 1.21 |

*Table S4. Coefficient and CI from model testing arousal-manipulation effects in adults, using all experimental trials.*
